# Conservative water management in the widespread conifer genus *Callitris*

**DOI:** 10.1093/aobpla/plt052

**Published:** 2013-11-13

**Authors:** Timothy J. Brodribb, David M. J. S. Bowman, Pauline F. Grierson, Brett P. Murphy, Scott Nichols, Lynda D. Prior

**Affiliations:** 1 School of Plant Science, University of Tasmania, Hobart 7001, Tasmania, Australia; 2 School of Plant Biology, The University of Western Australia, Crawley 6009, Western Australia, Australia; 3 Present address: University of Melbourne, Parkville 3010, Victoria, Australia

**Keywords:** Cavitation, drought, hydraulic, plasticity, water management.

## Abstract

Water management by woody species encompasses characters involved in seeking, transporting and evaporating water. Examples of adaptation of individual characters to water availability are common, but little is known about the adaptability of whole-plant water management. Here we use plant hydration and growth to examine variation in whole-plant water management characteristics within the conifer genus *Callitris*. Using four species that cover the environmental extremes in the Australian continent, we compare seasonal patterns of growth and hydration over 2 years to determine the extent to which species exhibit adaptive variation to the local environment. Detailed measurements of gas exchange in one species are used to produce a hydraulic model to predict changes in leaf water potential throughout the year. This same model, when applied to the remaining three species, provided a close representation of the measured patterns of water potential gradient at all sites, suggesting strong conservation in water management, a conclusion supported by carbon and oxygen isotope measurements in *Callitris* from across the continent. We conclude that despite its large range in terms of rainfall, *Callitris* has a conservative water management strategy, characterized by a high sensitivity of growth to rainfall and a delayed (anisohydric) closure of stomata during soil drying.

## Abstract (precis)

How plants manage their water use in seasonally dry environments is a major component of each individual species' ecology. We examined closely related species of a highly successful Australian conifer genus, *Callitris*, to determine whether species growing under contrasting climates showed adaptive specialization in the way they used water. Sampling 4 *Callitris* species growing across a large climatic range we found that each exhibited a similar strategy of linking growth very tightly with rainfall events, and surviving dry periods by resisting damage to their water transport system. This strategy is similar to the Junipers of the Northern Hemisphere, and requires a cavitation-resistant xylem.

## Introduction

For every gram of carbon fixed during photosynthesis in the leaves of land plants, several hundred grams of water are lost. This unfavourable exchange rate is sustainable by virtue of the fact that water is, on average, abundant on earth. However, huge geographical and temporal variation in the availability of water on land surfaces means that water stress is a fundamental limitation to the survival and productivity of most land plant species. As a consequence, efficient water use has been the subject of intense selective pressure throughout the evolution of vascular plants (Raven 1977, 2000; McAdam and Brodribb 2012). Thus, all plants have a ‘water management strategy’ that can be conceptualized as a combination of water extraction (root), water transport (xylem), water storage (capacitance) and water use (stomata) physiologies that determine the moisture availability required for a particular species to survive and grow (Sperry 2003). The water management strategy of plant species therefore encompasses a nexus of evolutionary trade-offs revolving around the competing interests of maximizing growth while conserving sufficient water to ensure survival (Cowan 1986).

Evolution has yielded considerable functional diversity in each of the components that define plant water management and this, combined with the stochastic nature of rainfall, leads to a large range of potentially successful strategies in any particular environment. In the driest plant communities, species with contrasting water management strategies commonly coexist; for example, slow-growing species with shallow roots, frugal water use and xylem resistant to water stress grow alongside vigorous species with deep roots and a water transport system with high conductivity but also a high vulnerability to water-stress-induced cavitation (Meinzer *et al.* 1999; Choat *et al.* 2012; Fu *et al.* 2012; Bucci *et al.* 2013). Even in rainforest communities there appears to be significant variation in water management strategies, apparently driven by interactions between water use and competition for light (Markesteijn *et al.* 2011) as well as the community phylogenetic structure and regional history (Blackman *et al.* 2012). Given that the most rapid and direct impacts of climate change upon global vegetation are likely to be upon rates of transpiration and soil moisture, it is critical that we are able to quantitatively define the water management strategy of any given species, and link this mechanistically to survival limits in terms of soil water availability.

In terms of water management, the conifer genus *Callitris* (Cupressaceae) represents a functional extreme. Typically a small shallow-rooted tree, the xylem of several *Callitris* species has been shown to resist enormous hydraulic tension (>8 MPa) before significant stem cavitation occurs (Brodribb *et al.* 2010), placing it alongside *Juniperus* as one of the most stress-resistant tree genera known (Willson *et al.* 2008; Pittermann *et al.* 2013). Extreme xylem physiology should theoretically allow *Callitris* species to continue to extract small quantities of water between rainfall events as soil water potentials become increasingly negative, by maintaining hydraulic connection with the soil at extremely low water potentials. *Juniperus* species growing in dry parts of the USA have similarly resistant xylem and in these species it is thought that an extended period of water extraction from relatively dry soils allows trees to maintain subsistence levels of photosynthesis and transpiration as plant tissues desiccate to extreme water potentials (West *et al.* 2008). The resilient strategy adopted by both *Callitris* and *Juniperus* affords benefits of a high ratio of water extraction per unit investment in root volume, while also enabling a rapid and efficient utilization of low-intensity rainfall events (Brodribb *et al.* 2012). Given its water-stress-resistant credentials, it is not surprising that *Callitris* is the dominant conifer genus in the predominantly dry continent of Australia, where its distribution crosses the length and breadth of the continent (Bowman and Harris 1995). Interestingly, however, not all *Callitris* species are restricted to dry habitats in Australia, and the genus thrives in monsoon climates as well as extending into rainforest communities in tropical and temperate Australia and New Caledonia (Jaffré 1995). The broad distribution of *Callitris* begs the question of whether this climatic breadth is due to functional plasticity in water management strategy.

In this study we investigate key aspects of the water management strategies within the genus *Callitris* to determine whether the genus can be characterized by a single conservative type, or whether there is evidence of functional plasticity that enables different species to adopt different strategies according to rainfall abundance. We combine data from three different scales of observation, including (i) detailed measures of seasonal variation in gas exchange and hydration of one of the most widespread *Callitris* species (*C. columellaris* F. Muell. (*sensu* Farjon 2005)) at one site with highly seasonal rainfall; (ii) long-term seasonal variation in growth and hydration in four species from four sites in each of the four ‘corners’ of Australia (*C. columellaris*, *C. preissii* Miq., *C*. *macleayana* F. Muell., *C. rhomboidea* Rich. & A. Rich.); and (iii) continent-wide sampling of carbon and oxygen isotope discrimination in foliage of one species (*C. columellaris)* across the country covering a wide range of rainfall and seasonality. We hypothesized that all *Callitris* species adopt a conservative water management strategy regardless of prevailing climate. To test this we used detailed sampling of gas exchange and water potential in *C. columellaris* to generate a model of water management in terms of plant hydration and stomatal control, and then applied this model to long-term measurements of *Callitris* species across Australia to determine whether a single model could adequately explain seasonal trends across the continent.

## Methods

### Variation in gas exchange and hydration in *C. columellaris*

**Figure 1.**
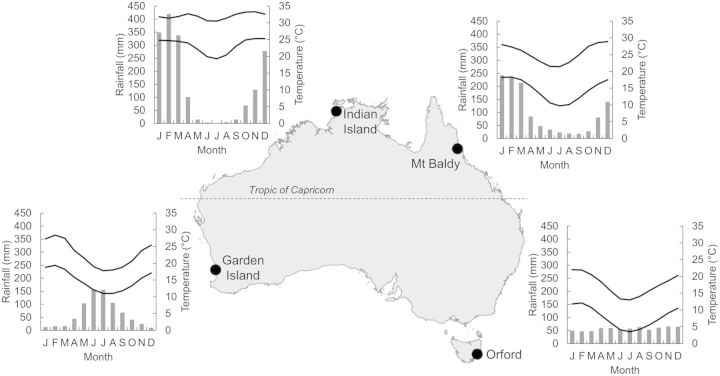
Location of the four *Callitris* monitoring sites throughout Australia. The seasonal distribution of mean monthly rainfall (vertical bars) and temperature (lines) for the four sites is shown.

Initially we conducted a detailed study of leaf gas exchange of *C. columellaris* (*sensu* Farjon 2005) upon which to construct a model of water management. Our study focused on a 60-year-old plantation of *C. columellaris* at Gunn Point (133.04°E, 12.25°S) in the monsoonal tropics of northern Australia (Bowman and Wightman 1985) adjacent to the long-term sampling site for *C. columellaris* at Indian Island (Fig. 1). *Callitris columellaris* is native to the region and this site was ideal because of ready access and very strong seasonality in rainfall, which enabled trees to be measured across a range of water potentials. After 60 years the plantation strongly resembles, both floristically and structurally, adjacent small remnant stands of long unburned *C. columellaris* (Bowman and Wightman 1985). Ten trees were tagged and every 4 months predawn and midday water potentials from crown leaves were measured. In addition, branches were sampled at midday and instantaneous measurements of photosynthetic gas exchange were made using a Li6400 portable photosynthesis system (LI-COR Biosciences, USA) with the cuvette set to ambient conditions of temperature, carbon dioxide (CO_2_) and humidity, and a light intensity of 1200 µmol quanta m^−2^ s^−1^ photosynthetically active radiation. Owing to the small size of individual leaves, small sprigs were sampled and the total leaf area in the cuvette was measured; fluxes are expressed per unit projected area. One shoot per tree was sampled from each of the tagged trees. Whole-plant hydraulic conductivity (*K*_plant_: mmol m^−2^ s^−1^ MPa^−1^) was calculated from the magnitude of the predawn–midday water potential difference (ΔΨ) and the transpiration rate (*E*) in individual branches.

A model to explain tree hydration in response to soil and evaporative conditions was formulated on hydraulic principles. The key driver of water flow through trees is the soil-to-leaf water potential gradient (ΔΨ) and hence this was designed as the model output, with input parameters of soil hydration (Ψ_predawn_) and leaf–air vapour pressure deficit (VPD). Based upon Ohm's law analogy,
(1)
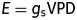

(2)
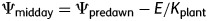

(3)
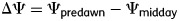

where stomatal conductance (*g*_s_) is a function of Ψ, and the soil-to-leaf hydraulic conductivity (*K*_plant_) is a function of Ψ_predawn_ (assumed to represent soil water potential). The empirical relationships between midday water potential and stomatal conductance, and predawn water potential and hydraulic conductivity were determined using data from the 10 experimental *C. columellaris* trees at Gunn Point in the Northern Territory (adjacent to Indian Island). We used this species and location because it experienced the greatest range of rainfall and water potential variation, thus allowing the broadest data set upon which to parameterize the hydraulic model. Inputs for the hydraulic model were Ψ_predawn_ and VPD, and wet season conditions for the *C. columellaris* population were simulated using humid soil (Ψ_predawn_ = −0.1 MPa) with a range of atmospheric VPDs from 0.6 to 3 kPa. Based on our observations and long-term climate data for Gunn Point (Bureau of Meteorology Australia), dry season conditions were simulated with ΔΨ_predawn_ declining from −0.1 to −6 MPa, with VPD ranging from 3 to 5 kPa.

### ‘Four corners’ sampling of four *Callitris* species

Four species growing at the four coastal extremities of Australia (Fig. 1) were examined over 2 years to determine whether they showed water management characteristics that were distinct or convergent with the water use behaviour characterized for *C. columellaris* above. The four sites chosen were:

1. Indian Island, northern Australia. This is a dense woodland of *C. columellaris*. The climate is monsoon tropical, with year-round high temperatures and high annual rainfall strongly concentrated in the summer months. Köppen–Geiger classification is ‘Tropical savanna with dry winter’ (Kottek *et al.* 2006).
2. Mt Baldy, north-eastern Australia. This is an open forest of *C. macleayana* and *Eucalyptus grandis*. The climate is monsoonal, though less intensely so than Indian Island, and slightly cooler due to its 950-m elevation. Köppen–Geiger classification is ‘Warm temperate with dry winter’.
3. Orford, south-eastern Australia. This is an open woodland of *C. rhomboidea* and *Eucalyptus pulchella*. The climate is cool maritime, with year-round rainfall. Köppen–Geiger classification is ‘Warm temperate, fully humid’.
4. Garden Island, south-western Australia. This is a low, dense woodland of *C. preissii*. The climate is classically Mediterranean, with a hot, dry summer and a cool, wet winter. Köppen–Geiger classification is ‘Warm temperate with dry summer’.

At each site 20 mature trees (diameter at breast height (DBH) ≥ 15 cm), representing a broad range of size classes, were permanently tagged and the location of each was recorded with a GPS. Trees that were obviously diseased or otherwise unhealthy were avoided. Selected trees were all within a short distance of each other (<500 m), and all within a relatively homogeneous habitat (i.e. without significant within-site variation in environmental variables such as soil type, slope, etc.).

Each of the 20 trees was fitted with a band dendrometer (ICT International, Armidale, NSW, Australia) at a height of 130 cm, and an initial dendrometer reading was taken. Miniature temperature and relative humidity sensors and data loggers (iButton DS1923, Maxim Integrated, San Jose, CA, USA) were attached to the southern side of each tree at a height of around 150 cm and set to record an observation every hour.

Over a period of 3 years and 3 months, the monitoring sites were visited quarterly, and readings were taken from the 20 band dendrometers. Leaf water potential was measured for each of the 20 trees 1–2 h before dawn and between 1200 and 1300 h.

As a measure of long-term (multi-year) integrated photosynthetic and water use characteristics, we examined the carbon and oxygen isotope levels in branches of trees. At each site, 5 of the 20 trees were selected for sampling foliar carbon and oxygen isotope concentrations. Foliage samples were placed in paper bags in the field, and then oven dried to constant weight at 60 °C. Finely ground subsamples were weighed into tin cups and analysed for δ^13^C using an automated nitrogen carbon analyser-mass spectrometer consisting of a 20/20 mass spectrometer connected to an ANCA-S1 preparation system (Europa Scientific Ltd, Crewe, UK) at the Western Australian Biogeochemistry Centre at the University of Western Australia. All samples were standardized against a secondary reference of radish collegate (41.51 % C; δ^13^C −28.61 ‰) that was subsequently standardized against primary analytical standards (IAEA, Vienna, Austria). Accuracy was measured as 0.07 % and precision as 0.03 %. For δ^18^O analysis, ∼0.25 mg subsamples were weighed into silver capsules and δ^18^O ratios were then measured using a high temperature conversion/elemental analyser (TC/EA) coupled with a Finnigan DELTA + XL mass spectrometer (Thermo Electron Corporation, Bremen, Germany). Internal lab standards for δ^18^O analysis were lab-sucrose (35.35 ‰, precision = 0.66 ‰) and lab-benzoic acid (20.05 ‰, precision = 0.41 ‰).

### Continental-scale sampling of ^13^C and ^18^O

We sampled the most widespread *Callitris* species (*C. columellaris*) to examine continent-wide patterns in stomatal behaviour across rainfall gradients. *Callitris columellaris* foliage was collected for ^13^C and ^18^O analysis from 90 sites across Australia, representing a wide range of climatic zones (arid, temperate and tropical), soil types, management regimes and disturbance histories that typified *C. columellaris* habitat within the region (Prior *et al.* 2011)*.* Some regions have extensive areas of *C. columellaris* forest or woodland, whereas others contain only small, isolated stands in fire-protected, rocky areas. We generally selected sites from among the larger stands in each region, based on information from local land managers, herbarium records and our observations as we drove through the region. At each site, three terminal sprigs of sun-exposed foliage were collected from the same height and positions around the canopy from each of five trees.

## Results

### *Callitris* tree growth in the ‘four corners’ of Australia

**Figure 2.**
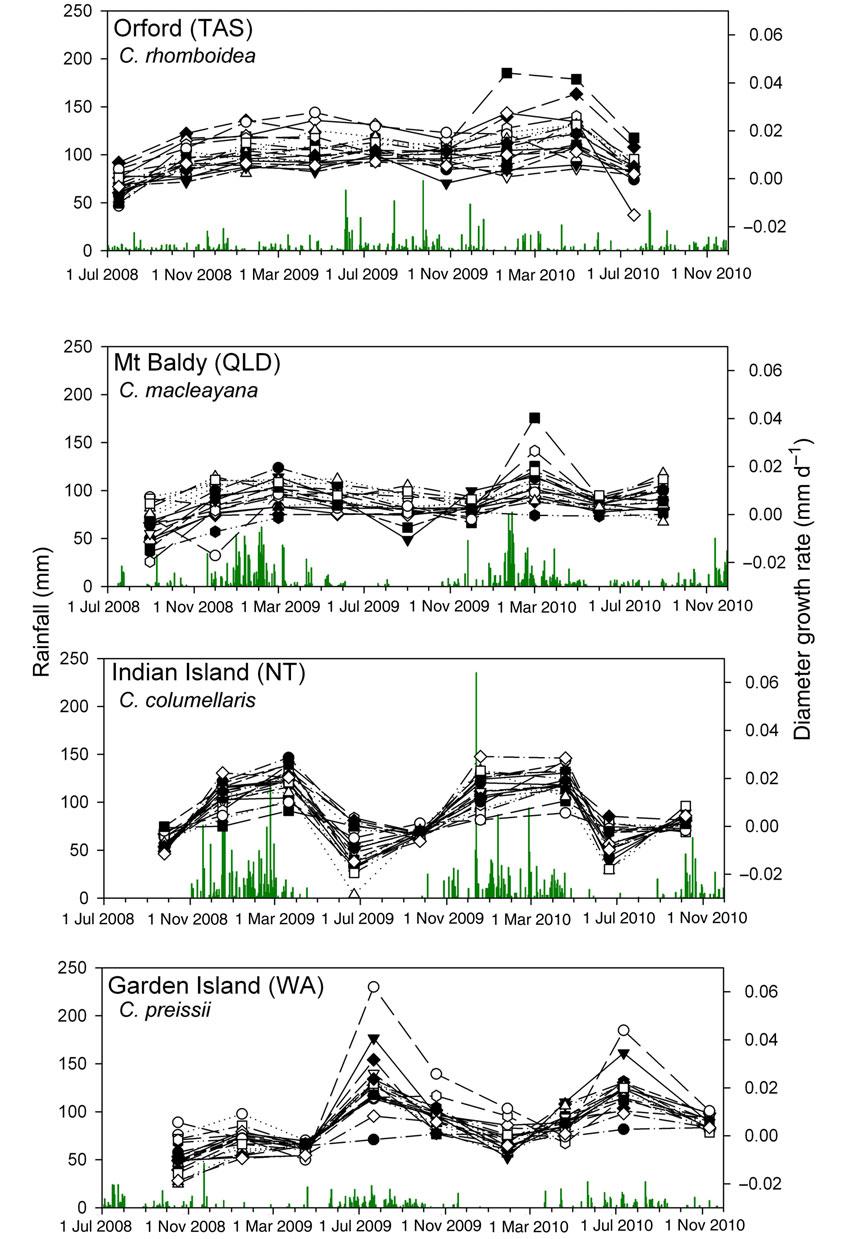
Patterns of growth (DBH) in the 20 banded trees over 2.5 years at each of the ‘four corners’ monitoring sites. Daily rainfall totals are shown as green bars for each locality. Each symbol represents an individual tree.

The seasonality of stem diameter growth varied considerably among sites, but with the exception of *C. rhomboidea* in Tasmania, growth was clearly responsive to seasonal fluctuations in rainfall. The magnitude of growth was less dependent on mean annual rainfall than the frequency of rainfall events, with growth rates decreasing quickly after the cessation of the wet season, regardless of whether winter/spring or summer dominant (Fig. 1). Hence mean cumulative growth in DBH over the period of the study was highest at Orford (*C. rhomboidea*, 7.0 mm over 2 years) where rainfall was relatively evenly distributed over the year, compared with lower growth in the seasonal rainfall at the tropical (*C. columellaris*, 4.01 mm; and *C. macleayana*, 4.36 mm) and Mediterranean (*C. preissii*, 3.77 mm) sites (Fig. 2).

#### Water potential model for *C. columellaris*

**Figure 3.**
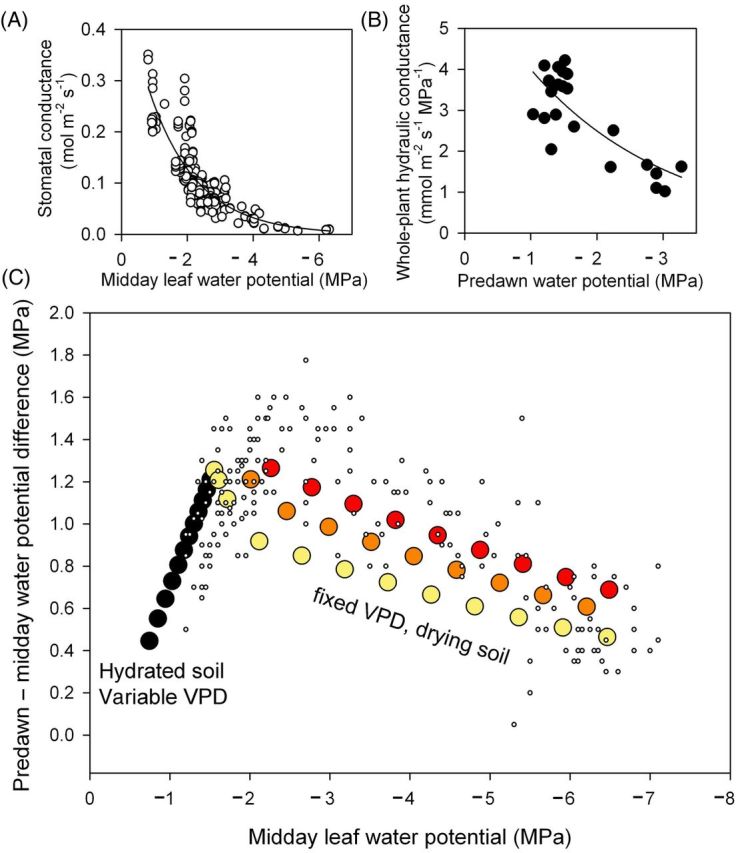
Relationships between midday leaf water potential and stomatal conductance (A) and predawn water potential and whole-plant hydraulic conductance (B) from seasonal measurements of 10 trees of *C. columellaris* measured adjacent to the Indian Island site in tropical northern Australia. These relationships were used in a hydraulic model in combination with the observed range of VPD and predawn water potential to generate the range of water potential gradients expected to develop during a typical year (C). Two phases are modelled: first a wet season scenario (black circles) with hydrated soil (predawn leaf water potential −0.1 MPa) and variable VPD (0.5–3 kPa), and second a dry season scenario with falling predawn water potential and fixed VPD at 3 kPa (yellow circles), 4 kPa (orange circles) or 5 kPa (red circles). These different modelled scenarios are compared with the observed data for Indian Island (small circles) and below for the other four corners site (Fig. 4).

**Figure 4.**
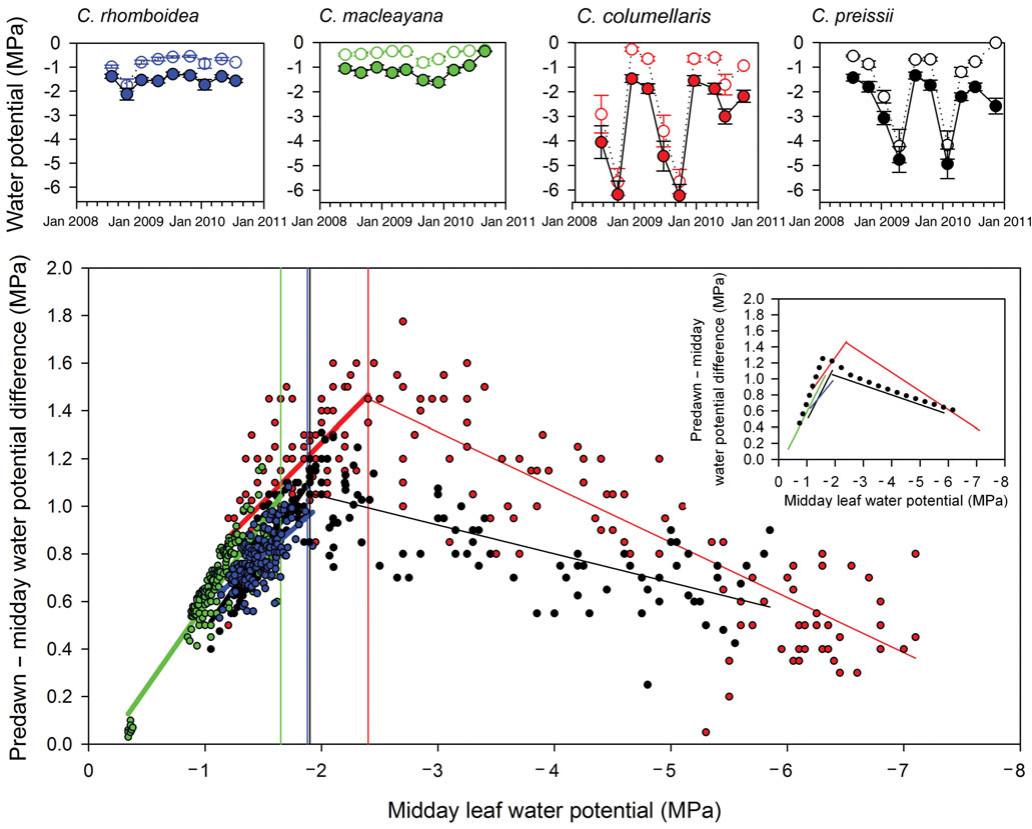
Seasonal trajectories of leaf water potential for each of the four species studied at the ‘four corners’ sites (top panels). Pooled water potential data (lower panel) show the relationship between midday leaf water potential and the whole-plant water potential gradient for each species, using the same species colour code as above. Transitions between positive and negative slopes were identified with LOESS curve fitting and are shown as vertical lines. Data fits (inset) for each species (straight lines using the same colour code) are compared with the modelled data for *C. columellaris* with VPD fixed as 3 kPa (dotted black line).

Given the strong dependence of growth on water availability, we sought to create a hydraulic model for *Callitris* gas exchange and hydration using *C. columellaris* trees at a highly seasonal site in the north of Australia (Gunn Point). This model was then compared with observed seasonal data at each of the ‘four corners’ sites mentioned above. Based upon seasonal measurements of gas exchange and water potential, we found that the stomatal response of *C. columellaris* was anisohydric (Fig. 3A), with stomatal conductance decreasing exponentially as Ψ_midday_ declined (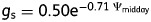; *r*^2^ = 0.82). Soil-to-leaf hydraulic conductivity was also highly sensitive to water potential (Fig. 3B), with a strong exponential decline in *K*_plant_ as Ψ_predawn_ became more negative (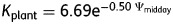). These empirical functions were used to define the standard model for *Callitris* with which to compare long-term water potential patterns found at the ‘four corners’ sites that encompassed a range of climates. We first tested the water potential model against data from *C. columellaris* at Indian Island and found that the model produced a similar pattern of Ψ_midday_ and ΔΨ to that observed in the field (Fig. 3C). Thus, in the range of Ψ_midday_ between 0 and −2 MPa, ΔΨ and Ψ_midday_ were linearly related until a peak value of ΔΨ was reached. Beyond this peak ΔΨ declined as Ψ_midday_ became more negative, driven by more negative predawn Ψ (Fig. 3C). Field measurements from the other three species of *Callitris* also displayed a close similarity to the patterns predicted by the hydraulic model (Fig. 4).

#### Seasonal water potential at the ‘four corners’ sites

Seasonal fluctuations in leaf water potential ranged widely between sites (Fig. 4), with very large ranges in mean midday water potential (Ψ_midday_) at the northern and western sites (−1.45 to −6.20 MPa and −1.42 to −4.89 MPa, respectively), while eastern sites, with less seasonal range in water availability, showed much diminished ranges (Ψ_midday_ water potentials above −2.1 MPa). Although Ψ_midday_ appeared to broadly track predawn water potential, ΔΨ (the difference between predawn and Ψ_midday_) showed distinctive patterns among species (Fig. 4). All species showed a strongly linear relationship between Ψ_midday_ and ΔΨ in the range of Ψ_midday_ between 0 and −1.5 MPa. Linear regressions fitted to each species in this range were not significantly different. In the two species where Ψ_midday_ fell substantially below −1.5 MPa, there was an abrupt transition from a positive slope between Ψ_midday_ and ΔΨ to a negative slope. Using a LOESS (locally weighted scatterplot smoothing) function in R it was possible to identify the transition between a positive and negative slope, which occurred at −1.89 MPa in *C. preissii* and at −2.4 MPa in *C. columellaris*. A slope transition was evident in *C. rhomboidea* at −1.90 MPa, but was difficult to identify in *C. macleayana* because minimum Ψ_midday_ only fell to around −1.60 MPa. Some variation between species was noted between the transition point from a positive to negative slope in the Ψ_midday_ versus ΔΨ relationship, but all species conformed well to the hydraulic model parameterized for *C. columellaris* (Fig. 4).

### Continental-scale leaf δ^13^C and δ^18^O

**Figure 5.**
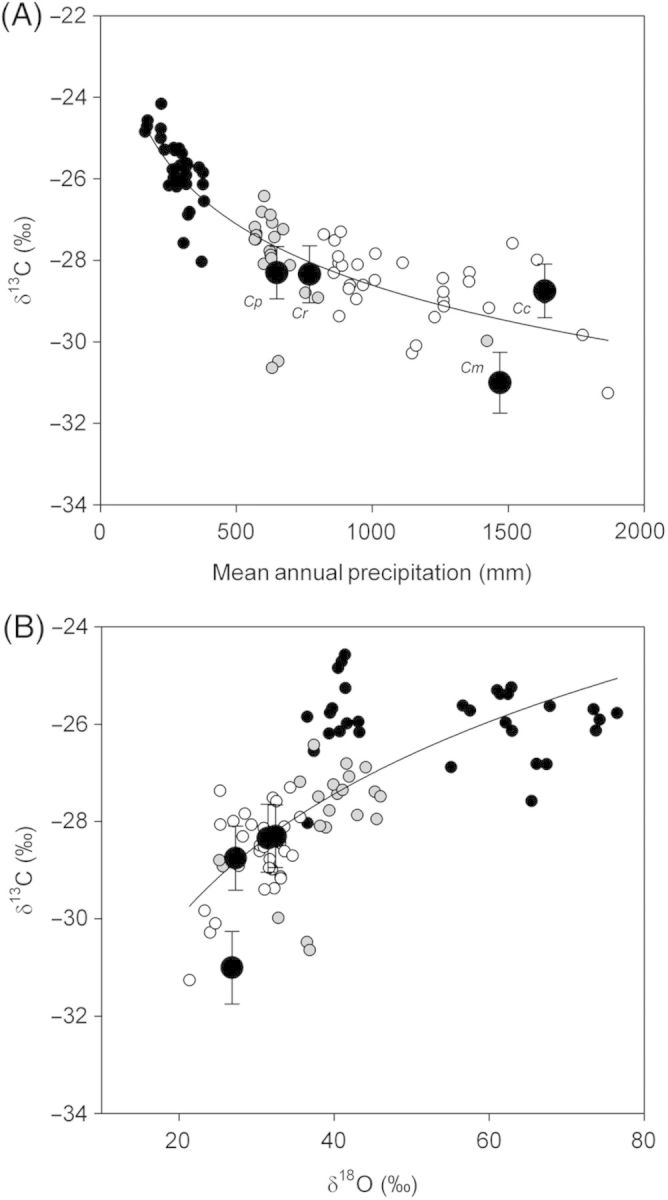
(A) Carbon isotope discrimination in shoots sampled from *C. columellaris* growing in dry (black), Mediterranean (grey) and tropical (open symbols) habitats in Australia, as well as the mean annual data for trees of *C. rhomboidea* *(Cr*), *C. preissi* (*Cp*), *C. columellaris* (*Cc*) and *C. macleayana* (*Cm*) from the four corners sites (large black symbols ± SD, *n* = 20). A strong correlation between δ^13^C and MAP is shown. (B) A comparison between δ^13^C and δ^18^O for the same plants as (A). Isotope discrimination of carbon and oxygen was strongly correlated in tropical and Mediterranean sites, but not in the dry sites.

Leaf δ^13^C values for all *Callitris* samples across species and sites showed a strong trend of decreasing discrimination with decreasing rainfall (Fig. 5). In the pooled data set there was a strong log relationship between leaf δ^13^C and site mean annual precipitation (MAP) (*r*^2^ = 0.74). Mean δ^13^C at the ‘four corners’ sites fell within the range of the continental *C. columellaris* data (Fig. 5). Leaf δ^13^C and δ^18^O were also strongly correlated across sites. However, as leaf δ^13^C becomes less negative, the relationship with δ^18^O is far more variable (at ∼δ^13^C > −28 ‰).

## Discussion

We found a consistent conservative strategy of growth and water use among *Callitris* species that spanned a large range of rainfall and temperature from across the continent of Australia. The hydration and growth of trees at all sites were highly dependent upon recent rainfall, leading to large fluctuations in growth and leaf water potential in regions with rainfall seasonality. A combination of anisohydric stomatal control, shallow roots and cavitation-resistant xylem appears to be common among *Callitris* species across Australia, making them highly successful opportunistic users of water. Interestingly, this strategy appears to be effective across a large precipitation range, including relatively mesic locations that experience more than 1500 mm of rainfall annually and are classified as rainforest.

The opportunistic nature of *Callitris* water use is clearly evidenced by a characteristic stomatal control pattern common to all sampled species. Based upon the dynamics of stomatal control in a seasonally dry stand of *C. columellaris*, we found that in this species, like other *Callitris* species (Attiwill and Clayton-greene 1984; Cullen *et al.* 2008; Brodribb and McAdam 2013), the sensitivity of stomata to desiccation was anisohydric. This means that stomatal control is weaker in *Callitris* than in isohydric conifers such as *Pinus*, where high sensitivity of stomata to desiccation leads to a conservative use of water by the maintenance of static midday leaf water potential in all but the most stressful conditions (Tardieu and Simonneau 1998). In anisohydric species subject to declining soil water availability, stomata tend to close gradually over a large range of water potentials, thereby leading to a ‘weaker’ containment of transpiration during the onset of water stress. The seasonal dynamics of predawn and midday leaf water potential in all species here showed a typical anisohydric pattern whereby midday leaf water potential was a function of soil water content (measured as predawn water potential) and transpiration rate (Fig. 3C). Recently it was shown that this type of stomatal behaviour was associated with declining levels of the ‘drought hormone’ abscisic acid (ABA) as water stress intensifies, thus reducing the sensitivity of stomata to leaf drying and prolonging stomatal closure during drought (Brodribb and McAdam 2013). Although this strategy potentially allows low levels of photosynthesis to be sustained during long periods of water stress (McDowell *et al.* 2008), another important feature of declining ABA levels during drought in *Callitris* is that stomata are able to open very quickly upon rehydration after prolonged water stress (due to low levels of ABA). The resultant very rapid recovery of photosynthesis and growth after periods of water stress must facilitate the opportunistic water-use strategy of *Callitris* (Brodribb and McAdam 2013).

Interactions between leaf hydration and stomatal control in all species here were compared by examining the maximum water potential gradient across trees at midday (ΔΨ). This parameter is of particular significance because it determines the water potential available to drive water movement through the tree, and is thus proportional to the transpiration rate at constant hydraulic conductance. When ΔΨ was plotted against midday water potential (Fig. 4), we found that all species conformed to a distinctive two-phase relationship, whereby midday leaf water potential (Ψ_midday_) was initially driven by ΔΨ (and thus by the rate of transpiration) in hydrated plants, but by soil water potential (Ψ_predawn_) in water-stressed plants. An abrupt transition between these two phases occurred when Ψ_midday_ decreased to between −1.9 and −2.4 MPa, at which point ΔΨ began to decline as water stress intensified. Declining ΔΨ occurred as Ψ_predawn_ fell to a point where stomatal conductance and transpiration began to drop, thus reducing the water potential gradient across the plant. This explanation for the dynamics of ΔΨ and Ψ_midday_ was confirmed in *C. columellaris* by parameterizing a simple hydraulic model of leaf water potential based upon empirically determined stomatal and hydraulic responses of whole trees (Fig. 3). Rendering water potential data in this way provides an excellent means of visualizing the water-use ‘strategy’ of any particular species in more detail than simply classifying species as isohydric or anisohydric. By integrating the effects of transpiration, hydraulic efficiency and stomatal conductance, it is possible to identify break points in the canopy response to soil drying as well as visualizing where a plant lies in the trajectory towards death by dehydration. Although the shape of the modelled responses closely matched the observed data for *C. columellaris*, the magnitude of ΔΨ was slightly higher in measured, as compared with modelled, plants. The most likely reason for this is that the simple exponential function fitted to the stomatal data (Fig. 3A) was an imperfect representation of stomatal function, and it is likely that the combination of transient changes in ABA, osmotic adjustment and a limited maximum stomatal aperture probably leads to a more logistic relationship between Ψ and stomatal conductance (Brodribb and Cochard 2009). Water potential data from the other three *Callitris* species in the four corners sites also conformed to a similar two-phase model of leaf hydration and ΔΨ, although the sites in Tasmania and tropical Queensland did not get dry enough to enter the second phase of declining ΔΨ.

A consistent relationship between foliar ^13^C isotope discrimination and rainfall in *Callitris* across the continent further supports our conclusion that stomatal regulation was conservative among *Callitris* sites and species (Fig. 5). Reduced ^13^C discrimination at the dry end of *Callitris* distribution would be an expected consequence of the shallow rooting strategy of the genus. At dry sites, where rainfall is typically sporadic, a higher proportion of photosynthesis and growth would be undertaken under drying atmospheric conditions after rainfall events. Stomatal sensitivity to humidity would lead to reduced stomatal aperture and a reduction of photosynthesis by diffusion-limited internal CO_2_ levels in the leaf, thus reducing ^13^C discrimination. A positive correlation between δ^13^C and δ^18^O among trees also points to stomatal control as being the major limiter of photosynthesis (Scheidegger *et al.* 2000; Cullen *et al.* 2008). However at the driest end of the range measured, there is a slight decoupling of the carbon and oxygen isotope relationship, which suggests that during extreme dry periods, photosynthetic capacity may be down-regulated by desiccation beyond the effects of lowered stomatal conductance. This finding supports the conclusion from our growth and water potential data that *Callitris* in Australia, or at least the four species examined, are all similarly responsive to rainfall. This conservative *Callitris* strategy of restricting photosynthesis and growth to wet periods and avoiding photosynthesis in dry months will lead to a highly efficient use of water during photosynthesis and growth in the long term, but must come at a cost to productivity.

Aspects of *Callitris* water management identified here contrast with observations for the most dominant tree genus in Australia, the broadleaf evergreen *Eucalyptus* and *Corymbias* (eucalypts), which typically co-occurs with *Callitris*. Unlike *Callitris*, the ^13^C discrimination of eucalypts appears unchanged across strong rainfall gradients (Schulze *et al.* 2006; Cernusak *et al.* 2011; Heroult *et al.* 2013), a feature that has been attributed at least in part to a deeper-rooting strategy (Janos *et al.* 2008; Heroult *et al.* 2013), coupled with a modification of leaf turgor relations (Poot and Veneklaas 2013), anatomy (Schulze *et al.* 2006) and area (Prior and Eamus 2000) to accommodate different soil water availability. In addition, it seems likely that stomata exercise stronger homeostatic control in eucalypts than *Callitris*, such that *Eucalyptus gomphocephala* growing near the Garden Island field site was found to maintain ΔΨ relatively constant throughout the year (Franks *et al.* 2007). By contrast we show here that *Callitris* species across a range of habitats had relatively insensitive stomata, leading to large but predictable seasonal variation in ΔΨ (Fig. 4). *Callitris* species appear to represent a strategic extreme, being shallow rooted and reliant on extremely cavitation-resistant xylem to maintain hydraulic integrity, but with low stomatal sensitivity to desiccation due to their declining levels of (stomatal-closing) ABA during sustained water stress (Brodribb and McAdam 2013).

It is interesting then to note the widespread coexistence of *Callitris* species alongside eucalypts with an entirely different, and potentially more adaptable, water management strategy. Such contrasts in the water management of competing evergreen trees have some parallel in the highly studied Piñon-juniper woodlands in the USA (Linton *et al.* 1998; McDowell *et al.* 2008), with *Callitris* adopting a similar strategy to its fellow Cupressaceae species *Juniperus osteosperma*, and *Eucalyptus/Corymbia* species adopting a similar water management and fire ecology role to that of *Pinus edulis*.

## Conclusions

Our data provide evidence of a water management strategy in *Callitris* that remains conserved across the continent, and which appears to contrast with that of the eucalypts (*Eucalyptus* and *Corymbia*) that dominate the Australian landscape. Co-occurrence of strongly contrasting water management strategies is relatively common, and it is of great interest and importance to understand the relative benefits of divergent management strategies. Doing so will provide the opportunity to understand the physiological basis for plant community assembly (Blackman *et al.* 2012) and response to change.

## Sources of Funding

This work was supported, in part, by several grants (Commonwealth Environment Facilities Research Fund Grant No. B0016193 (D.M.J.S.B.) and Australian Research Council Discovery Project Nos. DP0878177 (D.M.J.S.B.) and DP120101868 (T.J.B.)). T.J.B. was funded by an Australian Future Fellowship (FT100100237).

## Contributions by the Authors

T.J.B. wrote the paper with input from D.M.J.S.B., L.D.P., P.F.G. and B.P.M. The study was conceived by D.M.J.S.B., L.D.P., P.F.G. and T.J.B. Data were collected and analysed by B.P.M., S.N., P.F.G. and T.J.B.

## Conflicts of Interest Statement

None declared.
